# LILRB2 Interaction with HLA Class I Correlates with Control of HIV-1 Infection

**DOI:** 10.1371/journal.pgen.1004196

**Published:** 2014-03-06

**Authors:** Arman A. Bashirova, Enrique Martin-Gayo, Des C. Jones, Ying Qi, Richard Apps, Xiaojiang Gao, Patrick S. Burke, Craig J. Taylor, Jerome Rogich, Steven Wolinsky, Jay H. Bream, Priya Duggal, Shehnaz Hussain, Jeremy Martinson, Amy Weintrob, Gregory D. Kirk, Jacques Fellay, Susan P. Buchbinder, James J. Goedert, Steven G. Deeks, Florencia Pereyra, John Trowsdale, Mathias Lichterfeld, Amalio Telenti, Bruce D. Walker, Rachel L. Allen, Mary Carrington, Xu G. Yu

**Affiliations:** 1 Cancer and Inflammation Program, Laboratory of Experimental Immunology, Leidos Biomedical Research Inc., Frederick National Laboratory for Cancer Research, Frederick, Maryland, United States of America; 2 Ragon Institute of MGH, MIT and Harvard, Boston, Massachusetts, United States of America; 3 Department of Pathology, Cambridge University, Cambridge, United Kingdom; 4 Tissue Typing Laboratories, Addenbrookes Hospital, Cambridge, United Kingdom; 5 Northwestern University Medical School, Chicago, Illinois, United States of America; 6 Department of Molecular Microbiology and Immunology, Johns Hopkins Bloomberg School of Public Health, Baltimore, Maryland, United States of America; 7 Fielding School of Public Health, University of California at Los Angeles, Los Angeles, California, United States of America; 8 University of Pittsburgh, Pittsburgh, Pennsylvania, United States of America; 9 USU Infectious Disease Clinical Research Program, Bethesda, Maryland, United States of America; 10 Johns Hopkins University School of Public Health, Baltimore, Maryland, United States of America; 11 School of Life Sciences, Ecole Polytechnique Fédérale de Lausanne, Lausanne, Switzerland; 12 San Francisco Department of Public Health, San Francisco, California, United States of America; 13 Division of Cancer Epidemiology & Genetics, NCI, Bethesda, Maryland, United States of America; 14 University of California at San Francisco Medical School, San Francisco, California, United States of America; 15 Infectious Disease Division, Brigham and Women's Hospital, Boston, Massachusetts, United States of America; 16 Infectious Disease Division, Massachusetts General Hospital, Boston, Massachusetts, United States of America; 17 Institute of Microbiology, University Hospital and University of Lausanne, Lausanne, Switzerland; 18 Howard Hughes Medical Institute, Chevy Chase, Maryland, United States of America; 19 St George's Medical School, University of London, London, United Kingdom; Stanford University School of Medicine, United States of America

## Abstract

Natural progression of HIV-1 infection depends on genetic variation in the human major histocompatibility complex (*MHC*) class I locus, and the CD8^+^ T cell response is thought to be a primary mechanism of this effect. However, polymorphism within the *MHC* may also alter innate immune activity against human immunodeficiency virus type 1 (HIV-1) by changing interactions of human leukocyte antigen (HLA) class I molecules with leukocyte immunoglobulin-like receptors (LILR), a group of immunoregulatory receptors mainly expressed on myelomonocytic cells including dendritic cells (DCs). We used previously characterized HLA allotype-specific binding capacities of LILRB1 and LILRB2 as well as data from a large cohort of HIV-1-infected individuals (N = 5126) to test whether LILR-HLA class I interactions influence viral load in HIV-1 infection. Our analyses in persons of European descent, the largest ethnic group examined, show that the effect of *HLA-B* alleles on HIV-1 control correlates with the binding strength between corresponding HLA-B allotypes and LILRB2 (p = 10^−2^). Moreover, overall binding strength of LILRB2 to classical HLA class I allotypes, defined by the *HLA-A/B/C* genotypes in each patient, positively associates with viral replication in the absence of therapy in patients of both European (p = 10^−11^–10^−9^) and African (p = 10^−5^–10^−3^) descent. This effect appears to be driven by variations in LILRB2 binding affinities to HLA-B and is independent of individual class I allelic effects that are not related to the LILRB2 function. Correspondingly, *in vitro* experiments suggest that strong LILRB2-HLA binding negatively affects antigen-presenting properties of DCs. Thus, we propose an impact of LILRB2 on HIV-1 disease outcomes through altered regulation of DCs by LILRB2-HLA engagement.

## Author Summary

Leukocyte immunoglobulin-like receptors B1 and B2 (LILRB1 and LILRB2) bind HLA class I allotypes with variable affinities. Here, we show that the binding strength of LILRB2 to HLA class I positively associates with level of viremia in a large cohort of untreated HIV-1-infected patients. This effect appears to be driven by *HLA-B* polymorphism and demonstrates independence from class I allelic effects on viral load. Our *in vitro* experiments suggest that strong LILRB2-HLA binding negatively affects antigen-presenting properties of dendritic cells (DCs). Thus, we propose an impact of LILRB2 on HIV-1 immune control through altered regulation of DCs by LILRB2-HLA engagement.

## Introduction

HIV-1 disease progression is influenced by host genetic factors and varies greatly among infected individuals. Polymorphism in the *HLA* class I locus has been consistently shown to associate with HIV-1 infection outcomes by both the candidate gene approach [1] and genome-wide association studies [2], [3]. The influence of specific *HLA* class I alleles on HIV-1 disease is particularly obvious for *HLA-B* alleles, among which HLA-B*57 and -B*27 exhibit consistent protective effects [4], [5], [6], [7] and an allelic group called HLA-B*35-Px associates with accelerated disease progression [8].

*HLA* class I involvement in HIV-1 disease is primarily thought to be linked to cytotoxic CD8^+^ T lymphocyte (CTL) responses, which are restricted by the host's class I allotypes [9], [10]. However, alternative mechanisms may exist, given the fact that the HLA class I molecules represent important ligands for receptors regulating activities of innate immune cells. These include the killer cell immunoglobulin-like receptors (KIRs) and leukocyte immunoglobulin-like receptors (LILRs). Members of both receptor families have been implicated in anti-HIV immunity. For instance, certain combinations of *HLA-B* and *KIR3DL/S1* alleles encoding receptor-ligand pairs associate with slower disease progression, which may be due to increased natural killer cell responsiveness to infected cells [11], [12]. In addition, a strong LILRB2-HLA-B*35-Px interaction is suggested to impair dendritic cell (DC) function during HIV-1 infection, possibly leading to faster disease progression [13]. Down-modulation of DC function was also observed as a result of a stronger interaction between LILRB2 and HLA-B*27 loaded with the viral escape mutant KK10 L6M compared to the wild type peptide loaded complex [14].

LILRB1 and LILRB2 are the most well-studied members of the LILR family [15], [16]. These two receptors share 82% sequence homology and bind both classical and non-classical HLA class I molecules [17], [18]. LILRB2 is exclusively expressed on cells of the myeloid lineage, including conventional DCs, whereas LILRB1 can also be expressed by lymphoid cells. Upon ligand engagement, LILRB1 and LILRB2 induce inhibitory signals via immunoreceptor tyrosine-based inhibitory motifs (ITIMs) in their cytoplasmic tails. Thus, these inhibitory receptors, whose ligands are ubiquitously expressed, might play a role in elevating the activation threshold of the myelomonocytic cells and preventing self-damage. LILRB1/B2 interactions with HLA class I involve β2-microglobulin (β2m) and the α3 domain of the class I molecule, which are relatively conserved across allotypes [19], [20], [21]. A recent study demonstrated variability in binding of LILRB1- and LILRB2-Fc fusion proteins to individual class I allotypes, which included 31 HLA-A, 47 HLA-B and 16 HLA-C allotypes, indicating that additional regions of HLA class I molecules are involved in the interaction [22]. Compared to LILRB1, LILRB2 showed a greater degree of variability in binding to HLA allotypes. Notably, HLA-B*57:01 and -B*27:05, which associate with protection in HIV/AIDS, were among the weakest LILRB2 binders. Such a low binding level may reduce inhibitory effects of LILRB2 in DCs and thus contribute to the protective effect of the corresponding alleles.

Based on these findings, we hypothesized that the differential LILRB1/2-HLA binding may impact overall immune response to HIV-1 through modification of DC function and thus influence HIV-1 disease outcomes. Specifically, HLA molecules that bind more strongly to LILRB2 were predicted to blunt DC function, which may ultimately contribute to reduced immune control of viral replication and more rapid disease progression. To test this hypothesis, we used epidemiological and *HLA* genotyping data from several natural history cohorts of HIV-1-infected persons and analyzed clinical outcomes in these patients in relation to *in vitro* determined levels of interactions between individual HLA class I allotypes and LILRB1/B2. Our data suggest that the binding strength between LILRB2 and HLA may contribute to HIV-1 control.

## Results

### Effect of *HLA-B* on viral control correlates with the LILRB2 binding strength

**Figure 1:**
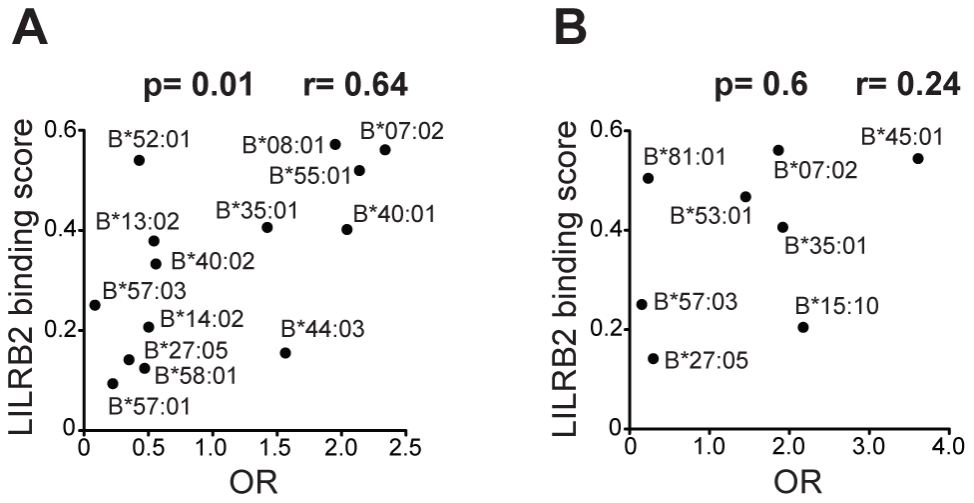
LILRB2 binding strength and odds ratios for viral load control for individual *HLA-B* alleles. The data plotted includes only alleles with significant association (p<0.05) for white (A) and black (B) patients in a univariate analysis for each *HLA-B* allele. Spearman correlation coefficient and p values are indicated.

To evaluate the influence of LILR-HLA interactions on HIV-1 disease, we tested for a potential correlation between LILR-HLA binding level and the strength of *HLA* allelic associations with viral control. Previously defined binding scores for HLA class I allotypes (Table S1) were used as a measure of LILR-HLA binding strength (Material and Methods, [22]). We compared the distribution of the *HLA* alleles in HIV-1 controllers and noncontrollers, all in the absence of therapy. Controllers were defined as individuals whose longitudinal mean viral load (mVL) remained below 2,000 copies per ml of plasma in the absence of therapy, whereas noncontrollers were patients whose mVL exceeded 10,000 copies per ml. Odds ratios (ORs) were calculated for each *HLA* allele using a univariate logistic regression model (Table S1), and significant ORs (p<0.05) were tested for correlations with the LILRB1/B2 binding scores in patients of European and African descent (referred to as whites and blacks, respectively). No relationship was found between the strength of LILRB1-HLA binding scores and the ORs of the corresponding alleles (Table S2). However, LILRB2 binding strength to HLA-B demonstrated a significant positive correlation with the ORs of the respective alleles in white patients (r = 0.64, p = 0.01; Table 1 and Figure 1). The correlation in our smaller cohort of black patients occurred in the same direction, but did not reach significance (r = 0.24, p = 0.6). Permutation analyses indicated that the significant positive correlation in whites is unlikely to have occurred by chance (p = 0.03). This finding suggests that the interaction between HLA-B and LILRB2 may participate in the overall effect of *HLA-B* alleles on HIV-1 control, where weaker binding of a given HLA-B allotype to LILRB2 correlates with greater protection of the corresponding allele, possibly as a consequence of enhanced DC function.

**Table 1:** Spearman correlation between LILRB2 binding strength and odds ratios of *HLA* alleles (p<0.05) for viral load control in HIV-1-infected individuals.

	N (alleles)	r	p
**Whites (N = 2685)**			
HLA-A	10	0.05	0.9
HLA-B	14	0.64	**0.01**
HLA-C	9	−0.38	0.3
**Blacks (N = 1306)**			
HLA-A	6	0.09	0.9
HLA-B	8	0.24	0.6
HLA-C	5	−0.30	0.6

### LILRB2 binding strength to HLA class I correlates with viral load in HIV-1 infection

**Figure 2:**
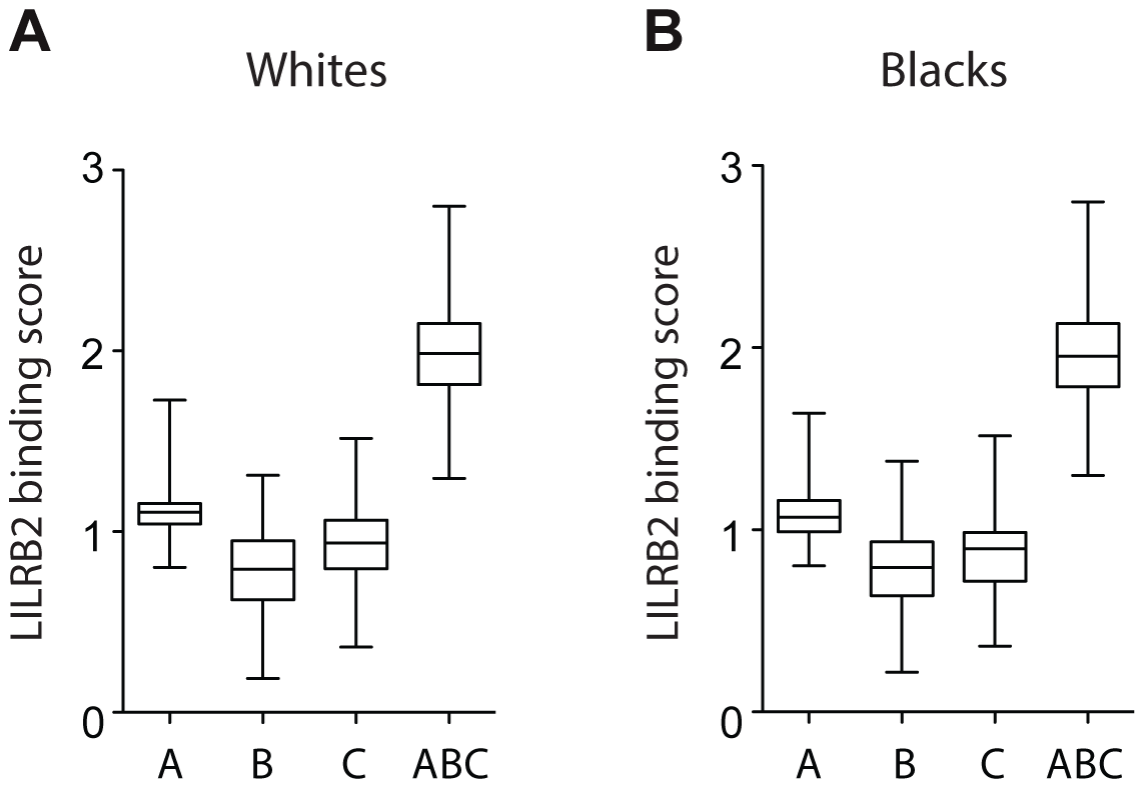
LILRB2-HLA binding score variations in 2900 white (A) and 1490 black (B) patients. A, B and C binding scores represent the sum of the binding scores for two alleles of the corresponding *HLA* class I locus. ABC binding score represents the sum of the locus-specific binding scores with the C scores counted at 1/10 level. Alleles with undefined scores were assigned the average of the scores for a given locus. Box and Whisker plots reflect median, the 25% and 75% percentiles and the minimum and maximum of all data.

A more rigorous test for an effect of LILRB2 on HIV-1 outcomes was performed by assigning to each patient four LILRB2-related scores, three locus-specific (A, B, C for HLA-A, -B and -C, respectively) scores and one combined (ABC) score, based on each patient's class I genotype, and then correlating these scores with measurements of HIV-1 disease outcomes in each patient. Locus-specific scores were generated as a sum of binding scores corresponding to the two alleles at each locus to reflect average LILRB2 binding. The combined ABC binding score was a sum of A, B and C scores, but for HLA-C, only 1/10 of the sum was incorporated into the final score since HLA-C is known to be expressed on the cell surface at roughly 1/10 the level of HLA-A and -B [23]. This combined score, which was used as a measure of average LILRB2 binding to class I on the cell surface, may be more relevant to the physiological consequences of the LILRB2 ligation than the locus-specific scores, since LILRB2 binds to all HLA-A, -B and -C allotypes [17], [22]. Notably, the variation in the B binding scores appears to be the main contributor to the variation of the ABC scores at the population level (Figure 2), given the relatively small range of the A scores and low 10% contribution of the C scores.

A significant positive correlation of the LILRB2-ABC binding scores with mVL used as a continuous variable was observed in both white and black patients using a univariate model (r = 0.21, p = 3×10^−30^ and r = 0.14, p = 5×10^−8^, respectively; Table 2). Locus specific analyses indicated that this correlation is driven mainly by the B scores (r = 0.24, p = 1×10^−38^ in whites and r = 0.16, p = 6×10^−10^ in blacks), since A scores show no significant correlation and C scores actually trend in the opposite direction, where higher binding of HLA-C to LILRB2 confers slight protection.

**Table 2:** Spearman correlation between LILRB2 binding strength and mVL in HIV-1-infected individuals.

	N	r	p
**Whites**	2900		
LILRB2-A		0.01	8E-01
LILRB2-B		0.24	**1E-38**
LILRB2-C		−0.12	**2E-10**
LILRB2-ABC		0.21	**3E-30**
**Blacks**	1490		
LILRB2-A		0.01	7E-01
LILRB2-B		0.16	**6E-10**
LILRB2-C		−0.01	6E-01
LILRB2-ABC		0.14	**5E-08**

To confirm that the LILRB2-HLA binding effect on HIV-1 disease outcomes is independent of the effects of individual class I alleles that are not related to LILRB2 binding, we used regression models with stepwise selection with p<0.05 as a threshold for inclusion, which included all class I alleles with phenotypic frequency of >2%, and LILRB2-HLA binding scores as continuous variables. The LILRB2-HLA binding effect on viral control was tested first in a categorical analysis comparing controllers to noncontrollers. The A, B and ABC binding scores demonstrated significant independent effects on viral control in white patients (OR = 1.2–1.3 for a change of 0.1 binding unit, p = 10^−3^–10^−18^; Table 3), whereas C score did not remain in the model. The B and ABC binding scores predicted viral control independently of all individual class I alleles in blacks as well (OR = 1.1–1.3 for a change of 0.1 unit binding, p = 10^−5^–10^−6^; Table 4), whereas the A and C binding scores did not stay in the model. Thus, the inverse correlation between the level of HIV-1 control and LILRB2 binding scores to HLA-B allotypes and to combined ABC allotypes were consistent in the two racial groups.

**Table 3:** Effect of LILRB2-HLA binding strength and individual class I alleles on viral control (controllers vs. non-controllers) in white patients.

Whites (N = 2685)											
LILRB2-A				LILRB2-B				LILRB2-ABC			
Covariate	p	OR	95%CI	Covariate	p	OR	95%CI	Covariate	p	OR	95%CI
B*57:01	2E-44	0.1	0.1–0.2	**LILRB2-B** 2	**3E-18**	**1.3**	**1.2-1.4**	B*57:01	7E-12	0.3	0.2–0.4
B*27:05	4E-15	0.3	0.2–0.4	A*01:01	2E-08	2.0	1.6–2.6	**LILRB2-ABC** 2	**9E-12**	**1.2**	**1.2–1.3**
A*01:01	5E-13	2.4	1.9–3.1	B*44:03	1E-07	2.8	1.9–4.1	A*01:01	1E-10	2.3	1.8–2.9
B*07:02	3E-07	2.0	1.5–2.6	B*57:01	1E-07	0.4	0.3–0.6	B*52:01	5E-07	0.3	0.2–0.5
B*13:02	6E-07	0.4	0.2–0.5	B*52:01	2E-07	0.3	0.2–0.4	C*04:01	5E-07	1.9	1.5–2.5
B*52:01	2E-06	0.3	0.2–0.5	A*25:01	3E-06	0.4	0.3–0.6	B*44:03	2E-06	2.6	1.7–3.8
B*14:02	3E-06	0.5	0.3–0.6	C*04:01	5E-06	1.8	1.4–2.3	B*13:02	3E-05	0.4	0.3–0.6
C*14:02	4E-05	0.4	0.2–0.6	B*40:01	5E-06	2.4	1.7–3.5	A*25:01	3E-05	0.4	0.3–0.7
A*25:01	7E-05	0.4	0.3–0.7	A*02:01	1E-04	1.5	1.2–1.8	B*40:01	4E-05	2.2	1.5–3.2
C*04:01	1E-04	1.6	1.3–2.1	B*49:01	2E-04	2.9	1.6–5.0	B*07:02	3E-04	1.7	1.3–2.2
A*02:01	5E-04	1.5	1.2–1.8	B*38:01	6E-04	2.4	1.5–3.9	A*02:01	4E-04	1.5	1.2–1.8
**LILRB2-A** 1	**7E-04**	**1.2**	**1.1–1.4**	C*07:02	9E-04	1.6	1.2–2.0	C*14:02	4E-04	0.4	0.2–0.7
B*40:02	9E-04	0.5	0.3–0.7	C*14:02	1E-03	0.4	0.3–0.7	B*49:01	9E-04	2.6	1.5–4.5
B*40:01	3E-03	1.8	1.2–2.6	A*31:01	2E-03	0.6	0.4–0.8	C*05:01	7E-03	1.5	1.1–1.9
A*24:02	2E-02	1.4	1.1–1.8	C*05:01	3E-03	1.5	1.1–2.0	A*68:01	8E-03	1.7	1.2–2.6
B*58:01	2E-02	0.5	0.3–0.9	B*13:02	4E-03	0.6	0.4–0.8	B*38:01	8E-03	2.0	1.2–3.2
B*18:01	2E-02	1.6	1.1–2.3	B*35:02	2E-02	3.3	1.3–8.9	A*24:02	2E-02	1.4	1.1–1.8
A*68:01	2E-02	1.7	1.1–2.5	A*68:02	5E-02	0.6	0.3–1.0	B*27:05	2E-02	0.7	0.5–0.9
B*15:01	4E-02	0.7	0.6–1.0					B*40:02	5E-02	0.6	0.4–1.0
								B*35:02	5E-02	2.7	1.0–7.0

Logistic regression model with stepwise selection included all *HLA* class I alleles with phenotypic frequencies of >2% and one of the A, B, C or ABC binding scores at a time. The results are shown for the p<0.05 cut-off. The C binding score did not stay in the model. ORs for binding scores reflect a change of 0.1 units.

1 stayed in the model with the p<0.01 cut-off but not with the p<0.001 cut-off.

2 stayed in the model with the p<0.01 and p<0.001 cut-offs.

**Table 4:** Effect of the LILRB2-HLA binding strength and individual class I alleles on viral control in black patients.

Blacks (N = 1306)							
LILRB2-B				LILRB2-ABC			
Covariate	p	OR	95%CI	Covariate	p	OR	95%CI
B*57:03	3E-14	0.3	0.1–0.3	B*57:03	6E-19	0.2	0.1–0.2
**LILRB2-B** 1	**5E-07**	**1.3**	**1.1–1.3**	A*23:01	4E-06	2.2	1.6–3.2
B*15:10	5E-05	6.2	1.9–6.2	**LILRB2-ABC** 2	**2E-05**	**1.1**	**1.1–1.2**
A*23:01	2E-04	2.7	1.4–2.7	B*39:10	3E-05	0.2	0.1–0.4
A*36:01	4E-04	9.9	1.9–9.9	A*36:01	3E-05	5.6	2.5–12.7
C*08:04	1E-03	0.6	0.1–0.6	B*81:01	3E-04	0.3	0.2–0.6
B*45:01	4E-03	4.3	1.3–4.3	B*15:10	5E-04	3.0	1.6–5.5
B*35:01	4E-03	2.8	1.2–2.8	B*35:01	1E-03	2.0	1.3–3.0
B*52:01	5E-03	0.8	0.2–0.8	B*45:01	2E-03	2.6	1.4–4.6
B*58:02	6E-03	4.2	1.3–4.2	B*58:02	4E-03	2.4	1.3–4.3
B*81:01	2E-02	0.8	0.2–0.8	C*08:04	1E-02	0.3	0.2–0.8
C*05:01	2E-02	0.9	0.3–0.9	B*52:01	2E-02	0.5	0.2–0.9
C*18:00	3E-02	0.9	0.3–0.9	A*68:02	2E-02	1.7	1.1–2.5
B*39:10	4E-02	1.0	0.1–1.0	A*02:02	5E-02	1.7	1.0–2.8
C*12:03	4E-02	1.0	0.2–1.0				

The analysis was similar to the one described in Table 3. The A and C scores did not stay in the model.

1 stayed in the model with the p<0.01 and p<0.001 cut-offs.

2 stayed in the model with the p<0.01 cut-off but not with the p<0.001 cut-off.

Next, we applied the linear regression model with stepwise selection to the analyses of mVL in the absence of therapy where mVL was a continuous variable. Among the four binding scores tested, the B and ABC scores showed significant positive correlations with mVL independently of the effects of individual class I alleles in both whites and blacks (Tables S3–S4). This analysis indicates that an increase in 0.1 unit of the ABC binding score would predict 0.08 and 0.03 log10 higher mVL in white and black patients, respectively, independently of individual *HLA* class I alleles. This translates to an increase of 1.1 and 0.5 log10 mVL in whites and blacks, respectively, when comparing patients with the highest ABC binding score to patients with the lowest score.

To test the stability of the regression models, we applied more stringent conditions in stepwise selection (p<0.01 and p<0.001 cut-offs). The B and ABC scores remained significant in the categorical analysis of viral load control in whites at both cut-offs (Table 3). While similar stability was observed for the B score in blacks, the ABC score remained significant in categorical analyses only at the intermediate cut-off (Table 4). In the continuous analysis of mVL, the binding scores demonstrated variable stability (Tables S3–S4).

Thus, we observed consistent associations for LILRB2-B and -ABC binding scores with HIV-1 control tested in both categorical and continuous analysis of mVL across the two racial groups. The effects were always less pronounced in the black population perhaps due to smaller number of individuals in this group. We also tested for a potential effect of LILRB2-HLA binding level on disease progression using a Cox model in a smaller cohort of seroconverts (780 whites and 287 blacks), but there was no significant effect on time to AIDS outcomes (see Materials and Methods) when individual class I alleles were included as covariables (data not shown). This negative result may be due to low statistical power, or the LILRB2 binding effect on HIV-1 control may be outcome-specific and influence viral load only.

### Functional effects of LILRB2-HLA interactions

**Figure 3:**
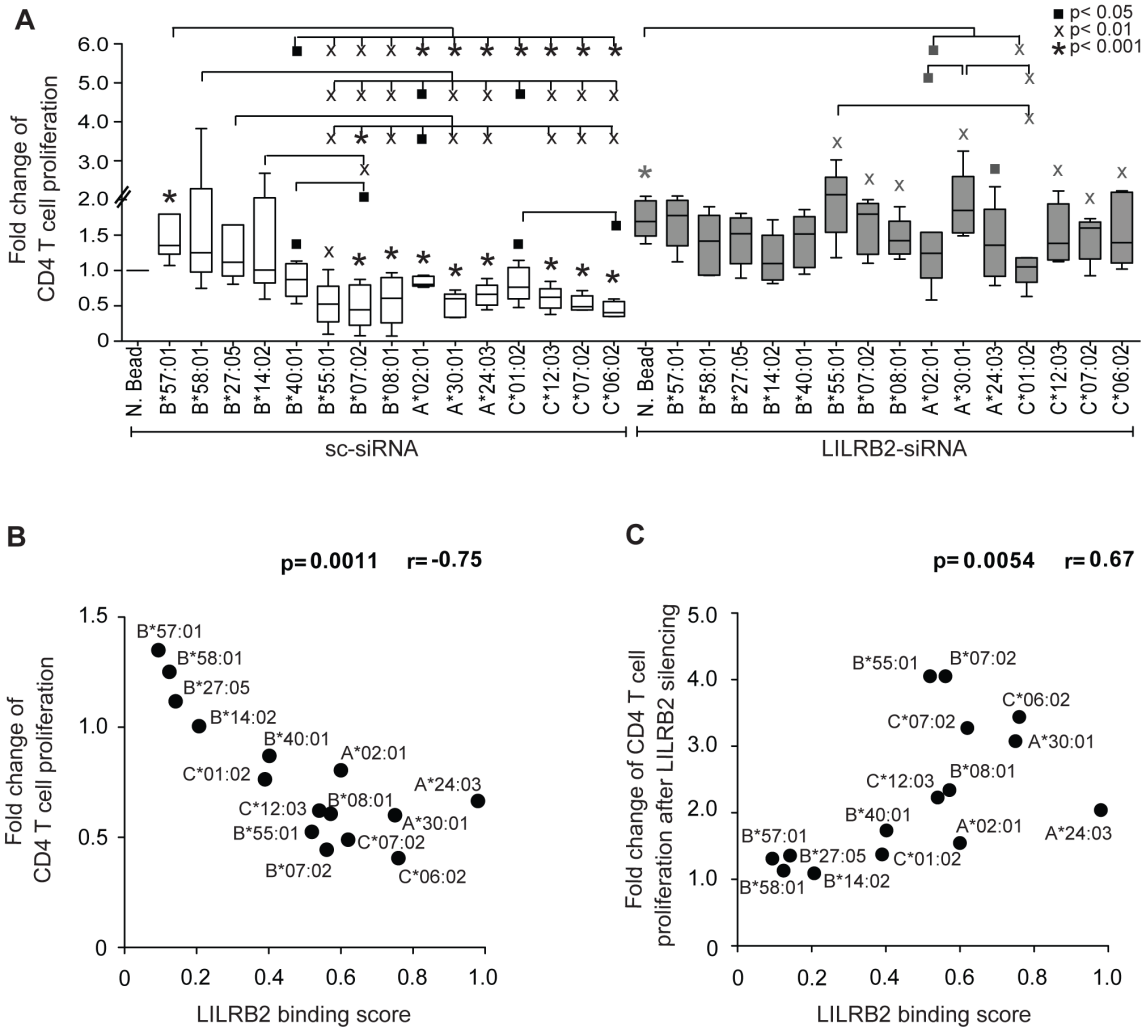
Impact of LILRB2-HLA interactions on functional properties of dendritic cells. (A) Fold changes in proliferative activities of allogeneic CD4^+^ T cells after exposure to MDDC treated with indicated HLA-A, -B or -C allotypes normalized to MDDC treated with negative beads (N. Bead), in the absence (white bars, n = 5, 8, 5 for HLA-A, -B, -C allotypes, respectively) or presence (gray bars, n = 5, 6, 5 for HLA-A, -B, -C allotypes, respectively) of siRNA-mediated downregulation of LILRB2 surface expression on MDDC. Significance was tested using one-way ANOVA followed by post-hoc analysis with the Tukey multiple comparison test, or using paired t-tests, as appropriate, (^▪^p<0.05, ^X^p<0.01, *p<0.001). (B): Spearman correlation between proliferative activities of allogeneic CD4^+^ T cells after incubation with MDDC treated with indicated HLA-A, -B and -C allotypes and corresponding LILRB2-HLA binding scores. (C): Spearman correlation between the ratios of MDDC function in the presence or absence of siRNA-mediated LILRB2 downregulation, and corresponding LILRB2-HLA-A, -B, -C binding scores.

Functional properties of DCs that result from altered LILRB2-HLA interactions were interrogated using mixed leukocyte reactions, an assay that measures the ability of DCs to stimulate antigen-specific T cell responses. Monocyte-derived dendritic cells (MDDC) were exposed to a panel of different recombinant HLA molecules, followed by cytokine-mediated maturation and incubation with CFSE-labeled allogeneic T cells according to a previously described protocol [24]. We observed divergent effects of different HLA allotypes on proliferative activities of allogeneic T cells, where the highest levels of proliferation were observed after MDDC exposure to HLA class I allotypes that have weakest binding to LILRB2, and the lowest proliferative activities were observed following exposure to HLA class I molecules with strongest binding to LILRB2 (Figures 3A and S2). These data are consistent with an inverse relationship between MDDC function and corresponding LILRB2-HLA binding strength (Figure 3B). siRNA-mediated knockdown of LILRB2 surface expression on MDDC (Figure S1) reversed inhibitory effects of HLA class I allotypes in a reciprocal hierarchical order (Figures 3A and S2), leading to a positive association between fold changes in MDDC function after LILRB2 knockdown and corresponding LILRB2-HLA binding scores (Figure 3C). However, inhibitory effects of two specific HLA class I allotypes (HLA-A*02:01 and -C*01:02) on DC function were not significantly affected by LILRB2 knockdown, suggesting that these HLA allotypes may interact with additional, as of yet unidentified immunoregulatory receptors on DCs. In contrast to antigen-presentation properties, secretion of TNFα, IL-6 or IL-12p70 by MDDC was not significantly influenced by LILRB2-HLA-B interactions (Figure S3). Together, these results suggest that LILRB2-HLA impact immune control of HIV-1 through alterations of the functional antigen-presenting properties of DCs.

## Discussion

Among all human *MHC* class I alleles, those encoded at the *HLA-B* locus have the highest degree of genetic variation and the dominant influence on HIV/AIDS [5]. Association of particular *HLA-B* alleles with HIV-1 infection outcomes is traditionally linked to the ability of the corresponding allotypes to elicit CTL responses. This concept is supported by numerous studies of HLA-restricted CTL responses and viral sequence evolution in carriers of specific *HLA* class I alleles [25]. The distinct effect of the *HLA-B* locus on cellular immune responses to HIV-1 is likely due to its greater level of diversity, which results in the presentation of a broader repertoire of viral peptides that can be presented by HLA-B allotypes as compared to HLA-A or HLA-C. In addition, relative resistance of HLA-B to downregulation by HIV-1 viral protein Nef compared to HLA-A [26] as well as low expression level of HLA-C were suggested to contribute to the principal role of the *HLA-B* locus in HIV-1 disease. However, the structural polymorphism of HLA-B can also influence its binding to receptors other than the T cell receptor. Based on the work presented herein, we propose that variation in binding properties of HLA-B to the inhibitory myelomonocytic receptor LILRB2 can contribute to the overall *HLA* effects on HIV-1 infection outcomes.

The ORs of individual *HLA-B* alleles determined by comparing HIV-1 controllers to noncontrollers correlate significantly with their LILRB2 binding strength in white patients (Figure 1A). A similar trend was observed in blacks, though not significantly so (Figure 1B), perhaps due to a smaller number of alleles considered in blacks (n = 8) as compared to whites (n = 14). B*81:01, an allotype present almost exclusively among blacks, appears to be an outlier in that it binds strongly to LILRB2, but associates with robust protection against HIV-1. B*81:01 contains an unusual polymorphism in the α3 domain that dramatically decreases CD8 binding (the same domain that is centrally involved in LILRB2 binding [27]), which may explain in part the protective role of the B*81:01 in HIV/AIDS [28], [29].

A more powerful and direct analysis of a correlation between LILRB2 binding scores and the level of viremia was conducted by assigning to each patient a LILRB2 binding score based on their class I genotypes and correlating these with the mVL determined from each patient. Our analyses included locus-specific (A, B, C) scores as well as a global ABC score, which was used as a measure of average LILRB2 binding to HLA class I overall. Highly significant correlations between mVL and B and ABC binding scores were observed in both white and black patients (Table 2). Two confounding factors may contribute to this strong correlation, including linkage disequilibrium between the *HLA* class I loci and the effects of individual *HLA* alleles on HIV-1 that are not related to LILRB2 function. Therefore, regression models with stepwise selection that included all individual class I alleles and LILRB2 binding scores were employed. The analyses indicated consistent effects for the B and ABC binding scores, both of which associated with viral replication tested in a categorical analysis (controllers vs. noncontrollers, Tables 3–4) and when mVL was used as a continuous variable in white and black patients (Tables S3–S4). Whereas the ABC score demonstrated effects similar to the B score, the B score accounts for all or nearly all of the combined ABC effect. The OR for viral control was 1.1–1.2 per 0.1 unit increase of the ABC binding score when comparing controllers to noncontrollers (Tables 3–4). It is not possible to compare directly the strength of the LILRB2 binding effect to the strength of individual *HLA* allelic effects since the former is based on a continuous variable (LILRB2 binding score) and the latter on a dichotomous variable (presence vs. absence of each allele). However, a comparison of the two patient groups at the extreme ends of the ABC binding scores (i.e. 10% of patients with the lowest scores vs. 10% of patients with the highest scores) results in an OR of 0.3–0.4, which is close to the strength of the protective effect of B*57 in the same model (OR = 0.2–0.3, Tables 3–4).

Neither the A nor the C binding scores demonstrated consistent effects on viral load when individual class I alleles were included in the model: the A score remained only in the categorical model when the least stringent p-value cut-off (<0.05) was used (Table 3), and the C score was not significant in any of the analyses. Thus, the negative correlation with mVL that was observed for the C scores in the univariate model (Table 2) is likely due to effects of individual alleles that are not related to LILRB2 binding and/or to linkage disequilibrium between *HLA-B* and -*C*.

Notably, the effects of B*27:05, B*57:01 and B*57:03 were substantially diminished in models that included LILRB2 binding scores relative to their effects in the absence of this covariable (Tables 3–4 and S3–S6). Alternatively, the effect of B*81:01 was not markedly influenced by inclusion of the binding scores in the model. These results indicate that the protective effects of the B*27 and B*57 alleles may be partially due to low LILRB2 binding, but this does not appear to be the case for B*81:01.

The correlation between HIV-1 immune control and the binding strength of LILRB2 to HLA-B allotypes specifically (and not HLA-A or -C) is difficult to comprehend, since LILRB2 binds all class I molecules without discrimination [22]. The substantially greater variation in binding scores for HLA-B as compared to HLA-A allotypes (Figure 2) may result in a greater influence of HLA-B on differential immune responses to HIV-1 across infected individuals. While HLA-C allotypes also show fairly broad variation in binding scores similar to HLA-B, their lower expression levels may diminish their effect in regulating myelomonocytic cells in HIV-1 infection. Alternatively, HLA-B expressed on the cell surface may behave in a distinct manner, for example due to the presence of intracellular cysteines as suggested by Gruda et al. [30]. Nevertheless, our model with combined ABC binding scores supports the idea that the average class I binding strength to LILRB2 can influence viral control, and the variation in this binding is mostly due to the allotypic diversity of the HLA-B binding strength to LILRB2.

The effect of LILRB2 binding to HLA class I on immune response to HIV-1 may be mediated by subsets of DCs expressing this receptor. Recent work demonstrated that dermal CD14^+^ DCs express both LILRB1 and LILRB2 [31]. These cells, along with Langerhans cells (LCs) and CD1a^+^ dermal DCs, are among the first immune cells encountered by HIV-1 in sexual transmission. Interestingly, CD14^+^ dermal DCs are less efficient at priming CTL than are LCs, and this difference has been attributed to the lack of LILRB1/2 expression by LCs [31]. The reduced ability of dermal CD14^+^ DCs to prime CTL was suggested to be due to competition between LILRB1/2 and CD8 in binding HLA class I, which has been demonstrated previously [27]. This competition may happen at the DC-T cell interface where LILRB molecules can interact *in cis* with HLA class I [32] on the DC surface, masking class I molecules from CD8 expressed by the T cells in a manner that does not necessarily involve inhibitory receptor signaling. Variation in the strength of LILRB2 binding to HLA class I may influence the capacity of dermal CD14^+^ DCs to prime virus-specific CTL and explain the effect of LILRB2 binding on viral load described herein. An alternative mechanism that is supported by our *in vitro* data implicates inhibition of DCs after LILRB2 ligation and receptor-mediated signal transduction. Our experiments demonstrate that stronger ligation of LILRB2 on the surface of MDDC by HLA *in trans* at an immature stage result in decreased capacity of these cells to stimulate T cell proliferation when they mature. This is in line with earlier work suggesting a regulatory role of the LILRB2 ligation in DC function [13], [14], [31], [33], [34]. Taken together, these data suggest that LILRB2-HLA interactions influence HIV-1 disease outcomes by regulating functional properties of DCs and their ability to generate antigen-specific T cell responses. Such effects are likely to be amplified by upregulation of LILRB2 surface expression on DCs in peripheral blood [35], [36] and lymph nodes [37] during progressive HIV-1 infection.

We have recently demonstrated a correlation between HLA-C expression level and HIV-1 control [38]. Analyses of *in vivo* CTL responses indicated that differential HLA-C expression influences CTL responses to HIV-1 peptides despite its lower overall cell surface expression relative to that of HLA-A and -B. The ability of HLA molecules, even those expressed at low levels, to trigger CTL killing of target cells is supported by *in vitro* data showing that as few as three HLA/peptide complexes can trigger CTL killing [39]. The mechanism of differential immune responses suggested in the current work is distinct from allotype-restricted CTL killing and involves regulation of DCs through engagement of LILRB2 with all allotypes of HLA-A, -B and -C (i.e. it is not allotype specific, distinguishing it from CTL killing). Due to the relatively low amount of HLA-C on the cell surface, the variation in its expression level would contribute minimally to the diversity of LILRB2 binding to HLA class I as a whole. Thus, differential HLA-C expression level has a significant effect on HLA-C-restricted CTL responses [38], but the overall low expression of HLA-C compared to HLA-A and -B limits its relative importance in mediating a response through LILRB2, which binds (at various levels) to all class I molecules.

The data presented herein underscore the complexity of HLA class I involvement in control of HIV-1 that goes beyond peptide presentation to CD8^+^ T cells. We propose that the LILRB2-HLA class I interaction may contribute to the effect of class I on HIV/AIDS through regulation of DC function. The relative size of this effect compared to the CTL or NK cell responses requires further investigation.

## Materials and Methods

### Study subjects

We used data from a total of 5126 HIV-1-infected individuals from eight US and one European cohorts: the AIDS Linked to Intravenous Experience (ALIVE), the U.S. military HIV Natural History Study (DoD HIV NHS), the DC Gay Cohort Study (DCG), the Multicenter AIDS Cohort Study (MACS), the Multicenter Hemophilia Cohort Study (MHCS), the Massachusetts General Hospital Controller Cohort (MGH), the San Francisco City Clinic Cohort (SFCCC), the Study on the Consequences of Protease Inhibitor Era (SCOPE) and the Swiss HIV Cohort Study (SHCS). Patients from MACS, MGH, SCOPE and SHCS, including 2685 white and 1306 black patients, were categorized in controller and noncontroller groups for the analysis of *HLA* class I impact on HIV-1 immune control. Longitudinal viral load data were available for 2900 white and 1490 black patients from ALIVE, MACS, MGH, DoD HIV NHS, SCOPE and SHCS. Seroconversion time and AIDS progression data were known for 780 white and 287 black patients from ALIVE, DCG, MHCS and SFCCC.

### Ethics statement

This study was approved by the protocol review office of the US National Cancer Institute institutional review board, as well as by the institutional review board of Massachusetts General Hospital. Informed consent was obtained at the study sites from all individuals. Patients' ethnicities were defined based on self-report.

### *HLA* genotyping

We performed genotyping of the *HLA-A/B/C* following the PCR-SSOP (sequence-specific oligonucleotide probing) typing protocol and PCR-SBT (sequence based typing) recommended by the 13^th^ International Histocompatibility Workshop (http://www.ihwg.org). All *HLA* class I genotypes were defined to 4-digit resolution with the exceptions of A*74:01/2, C*17 and C*18, which were determined to 2-digits.

### LILRB2-HLA binding scores

LILRB2-HLA binding scores were defined in a previously described experiment [22]. Briefly, a set of LILR-Fc fusion proteins was tested for binding with LABScreen HLA class I SABs at a concentration of 0.5, 1 and 2 µM. The level of binding was assessed by measuring the median fluorescence intensity (MFI) of the LILR-Fc bound to the beads using appropriate normalizations, which included subtraction of the Fc-negative control MFI and division of the result by the MFI of W6/32 (monoclonal anti-HLA class I antibodies recognizing β2m-associated HLA molecules). The normalized values were assigned to each HLA allotype as binding scores. Each binding score is a function of avidity of bivalent LILRB2-Fc for HLA, which in turn depends on the affinity of monomeric LILRB to HLA. Therefore, the binding score can be used as a quantitative characteristic of the strength of LILR-HLA interactions. The relative LILR binding to different HLA allotypes was similar at each of the LILR concentrations tested (Figure S4). This consistency between LILR concentrations assured us that the difference in MFIs between the allotypes is mainly due to difference in binding strength, and is not an experimental artifact. The 1 µM concentration results were chosen as a representative dataset. Among the HIV-1-infected patients used for the analyses, frequencies of the *HLA-A/B/C* alleles with unknown binding scores were 2/11/24% in white and 8/13/39% in black patients. To avoid power loss, we used mean values for the corresponding locus for each genotype with unknown score. The pairs of alleles A*74:01/2 and B*81:01 differ only at the signal peptide, therefore, they were treated as individual alleles in the context of LILRB2 binding.

### Mixed leukocyte reactions

Monocyte Derived Dendritic Cells (MDDC) were prepared as described previously. Briefly, 2 × 10^8^ PBMCs were plated in 5% pooled human serum medium and incubated during 60 min at 37°C to adhere monocytes. After discarding non-adherent cells, monocytes were differentiated into MDDC in the presence of RPMI 1640 medium supplemented with 50 µg/ml of GM-CSF (Amgen) and 10% fetal bovine serum. On day 5, immature MDDC were gently detached using PBS with 0.5% BSA and 2 mM EDTA, harvested and plated at 4x10^5^ cell/well in a round-bottom 96-well plate (Costar). Next, cells were incubated with beads coated with selected HLA-B allotypes, or uncoated control beads (One Lambda) for 30 min at 37°C, washed, and subsequently matured in the presence of a previously described cytokine cocktail containing 5 ng/ml IL-1β, 5 ng/ml TNFα, 1 µg/ml PGE-2 and 0.15 µg/ml IL-6. After 16 hours, mature MDDC were mixed with negatively-isolated CFSE-labeled allogeneic T cells at a DC:T cell ratio of 1∶100 for mixed lymphocyte reactions. Allogeneic T cell proliferation was determined after 6 days in culture by investigators blinded towards the added HLA class I molecules, using an LSRFortessa flow cytometer (Becton Dickinson).

### Cytokine secretion assays

To analyze cytokine secretion, immature MDDC were prepared and treated with HLA class I molecules as described above and then matured using 5 µg/ml CL097 (InvivoGen) in the presence of 5 µg/ml brefeldin A. After 20 hours, cells were fixed and permeabilized, stained with antibodies recognizing intracellular IL-12p70, TNFα and IL-6, and processed to flow cytometric acquisition by investigators blinded towards the added HLA class I allotypes.

### siRNA-mediated gene knockdown

10^6^ MDDC were suspended in 300 µl Optimem (Gibco) in the presence of 2 nmol of either LILRB2-specific (LILRB2-siRNA) or scramble control siRNA (sc-siRNA) pools (On-TARGET plus SMARTpool, Dharmacon) and transferred to a 4-mm electroporation cuvette (Bio-Rad Laboratories). Cells were left on ice for 10 min, electroporated (900 V, 0.75 msec square wave; Genepulser Xcell; Bio-Rad Laboratories), and transferred back to culture medium for another 24 to 48 hours. Efficiency of specific siRNA-mediated LILRB2 knockdown was determined by flow cytometry using an anti-LILRB2 antibody (clone 42D1, Biolegend).

### Statistical analysis

We used SAS 9.1 (SAS Institute) for data management and statistical analyses. The effect of *HLA* alleles on viral control was determined by categorical analysis of the allelic frequencies in HIV-1 controllers and noncontrollers. Corresponding ORs were calculated using logistic regression model with SAS procedure PROC LOGISTIC. Relationships between viral loads and LILRB2-HLA binding scores were analyzed by the Spearman correlation test using PROC CORR. Permutation analysis was done by random assignment of binding scores to *HLA-B* alleles (10,000 times) and testing the probability of significant Spearman correlation of the binding scores with ORs with p<0.05.

LILRB2 binding scores as continuous variables and presence versus absence of all individual *HLA* class I alleles of frequency ≥2% were included with stepwise selection in all regression models. Results in the tables are for the models using a threshold of a two-sided p value <0.05 for inclusion of a covariate as a significant independent effect. The stability of regression models was tested using more stringent thresholds of p<0.01 and p<0.001 for inclusion in the model. The results for the binding scores are indicated in the footnotes to the tables.

Cox proportional hazards model was applied to perform AIDS progression analysis by using PROC PHREG. For this, we estimated the seroconversion date as the midpoint between the first positive and the last negative HIV-1 antibody test (mean interval, 0.79 years; range, 0.07 to 3.0 years). Four end points reflecting disease progression (AIDS outcomes) were evaluated: time to CD4<200 cells/ml; progression to AIDS according to the 1987 definition by the Centers for Disease Control and Prevention (CDC, [40]); progression to AIDS according to the 1993 definition by CDC; and AIDS-related death [41].

Data of *in vitro* experiments were presented as Box and Whisker plots, reflecting the median, minimum, maximum and the 25^th^ and 75^th^ percentiles. Significance was tested using one-way ANOVA followed by post-hoc analysis with the Tukey multiple comparison test, or using paired t-tests, as appropriate.

## Supporting Information

Figure S1 siRNA-mediated downregulation of LILRB2 on monocyte-derived dendritic cells. Histogram reflects LILRB2 surface expression 48 hours after transfection with LILRB2-specific or control siRNA.
(PDF)

Figure S2 Impact of LILRB2-HLA interactions on functional properties of dendritic cells. Representative dot plots reflecting proliferative activities of allogeneic CD4^+^ T cells after incubation with MDDC exposed to indicated HLA-A (A), -B (B) and -C (C) allotypes, in the absence or presence of siRNA-mediated knockdown of LILRB2 surface expression. Numbers on dot plots indicate the proportion of proliferating CD4^+^ T cells.
(PDF)

Figure S3 HLA-B allotypes do not differentially affect cytokine secretion of MDDC. Data reflect proportions of MDDC secreting the indicated cytokines after exposure to different HLA-B molecules. Cumulative results from 4 independent experiments are shown.
(PDF)

Figure S4 The relative LILR binding strength to different HLA allotypes was similar at each of the LILR concentrations tested. Spearman correlations between the binding scores of 3 different tested concentrations of LILRB2 to HLA-A (A), -B (B) and -C (C) allotypes. Spearman correlation coefficient and p values are indicated on graphs. (D) Analysis of LILRB2 binding scores at the concentrations of 0.5, 1 and 2 µM to protective (blue) and high risk (red) HLA-B allotypes.
(PDF)

Table S1 *HLA* class I allele-specific LILRB1 and LILRB2 binding scores and corresponding odds ratios (OR) for viral load control as determined in a univariate model for each corresponding allele. ORs were not defined for some *HLA* alleles that cannot be genotyped (C*17:01 and C*18:02) and for alleles that were not present in the controller groups or in the whole population. Analysis for the combined cohort (All) was adjusted for race.
(PDF)

Table S2 Spearman correlation between LILRB1 binding strength and odds ratios (p<0.05) for viral load control in HIV-1-infected individuals.
(PDF)

Table S3 Effect of the LILRB2-HLA binding strength and individual class I alleles on mVL in white patients. Linear regression models with stepwise selection included all *HLA* class I alleles with phenotypic frequencies of >2% and one of the A, B, C or ABC binding scores at a time. The results are shown for the p<0.05 cut-off. The A and C scores did not stay in the model.
(PDF)

Table S4 Effect of the LILRB2-HLA binding strength and individual class I alleles on mVL in black patients. The analysis was similar to the one described in Table S3. The results are shown for the p<0.05 cut-off. The A and C scores did not stay in the model.
(PDF)

Table S5 Effect of individual class I alleles on viral control (controllers vs. noncontrollers). Logistic regression model with stepwise selection included all *HLA* class I alleles with phenotypic frequencies of >2%. The results are shown for the p<0.05 cut-off.
(PDF)

Table S6 Effect of individual *HLA* class I alleles on mVL. Linear regression model with stepwise selection included all *HLA* class I alleles with phenotypic frequencies of >2%. The results are shown for the p<0.05 cut-off.
(PDF)
