# Supplementary figures and images for: LILRB2 Interaction with HLA Class I Correlates with Control of HIV-1 Infection
Source: PLoS Genet. 2014 Mar 6;10(3):e1004196. doi: 10.1371/journal.pgen.1004196 (PMC3945438; doi:10.1371/journal.pgen.1004196)

Figure S1

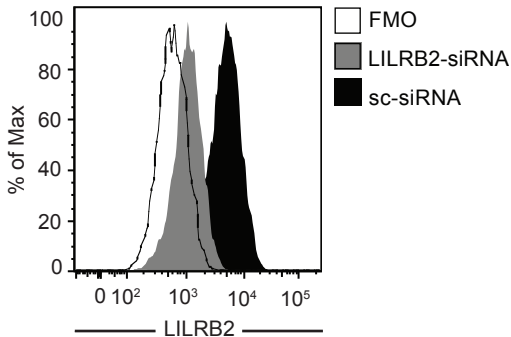

Supplement: Figure S1 — siRNA-mediated downregulation of LILRB2 on monocyte-derived dendritic cells. Histogram reflects LILRB2 surface expression 48 hours after transfection with LILRB2-specific or control siRNA. (PDF) [file pgen.1004196.s001.pdf]

Figure S2

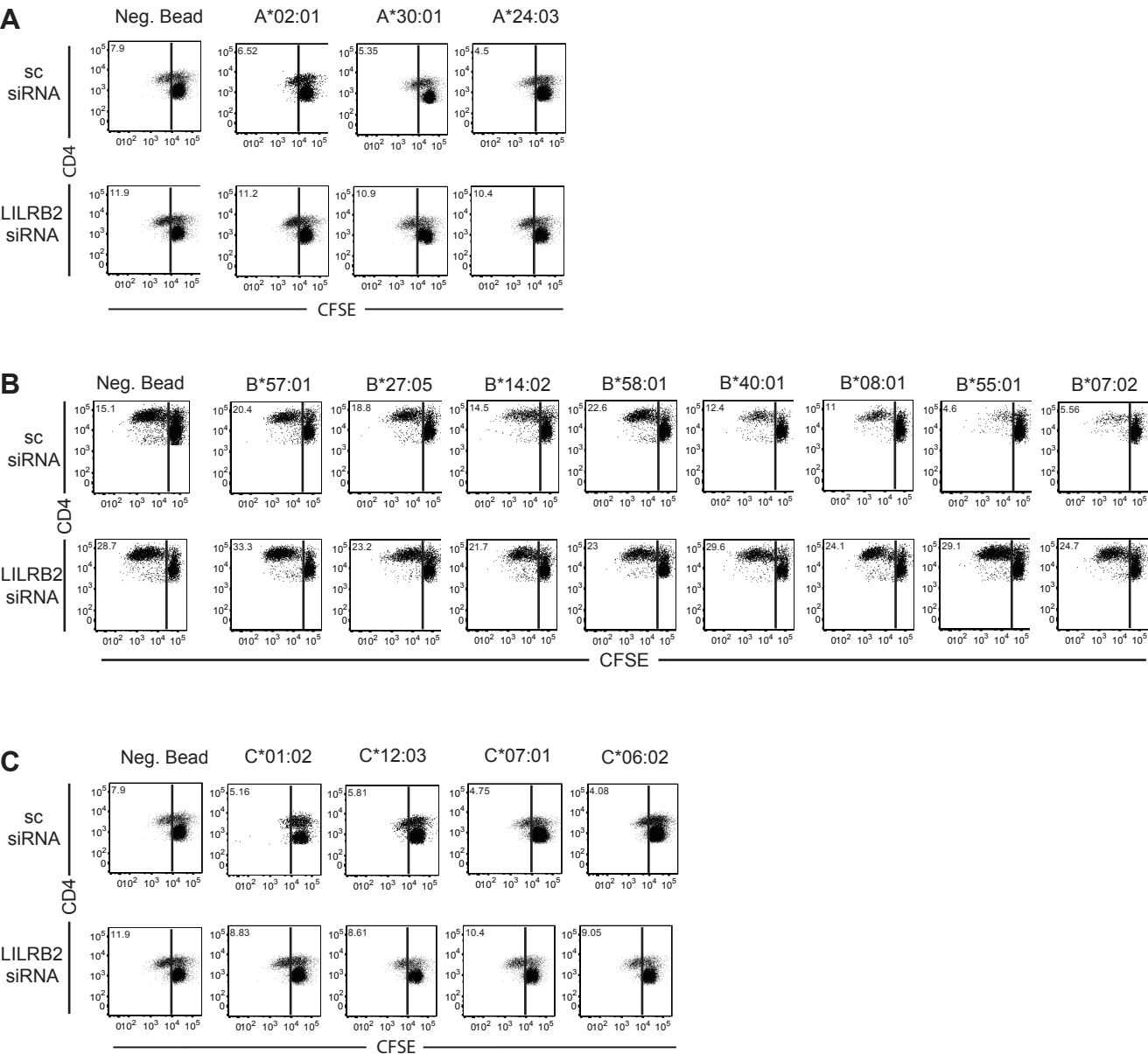

Supplement: Figure S2 — Impact of LILRB2-HLA interactions on functional properties of dendritic cells. Representative dot plots reflecting proliferative activities of allogeneic CD4+ T cells after incubation with MDDC exposed to indicated HLA-A (A), -B (B) and -C (C) allotypes, in the absence or presence of siRNA-mediated knockdown of LILRB2 surface expression. Numbers on dot plots indicate the proportion of proliferating CD4+ T cells. (PDF) [file pgen.1004196.s002.pdf]

Figure S3

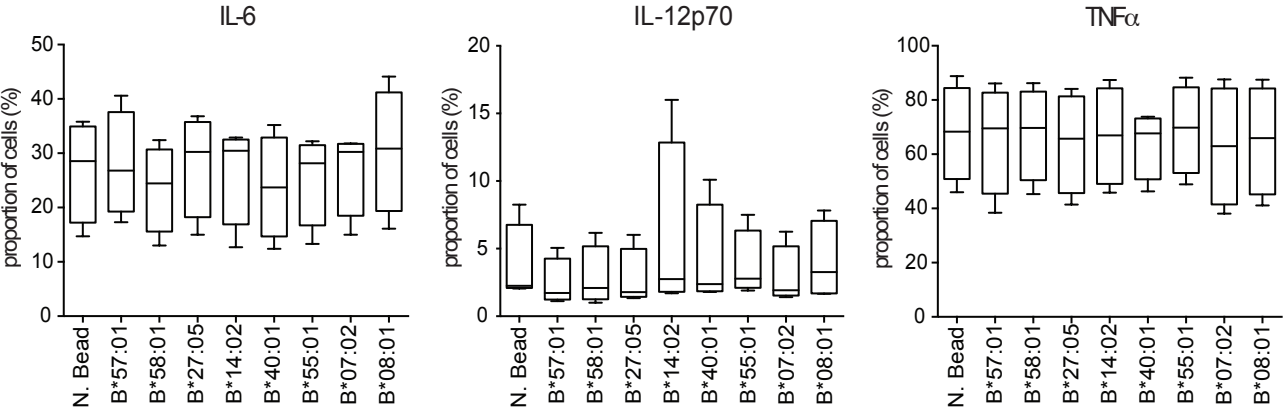

Supplement: Figure S3 — HLA-B allotypes do not differentially affect cytokine secretion of MDDC. Data reflect proportions of MDDC secreting the indicated cytokines after exposure to different HLA-B molecules. Cumulative results from 4 independent experiments are shown. (PDF) [file pgen.1004196.s003.pdf]

Figure S4

**A**

HLA-A alleles

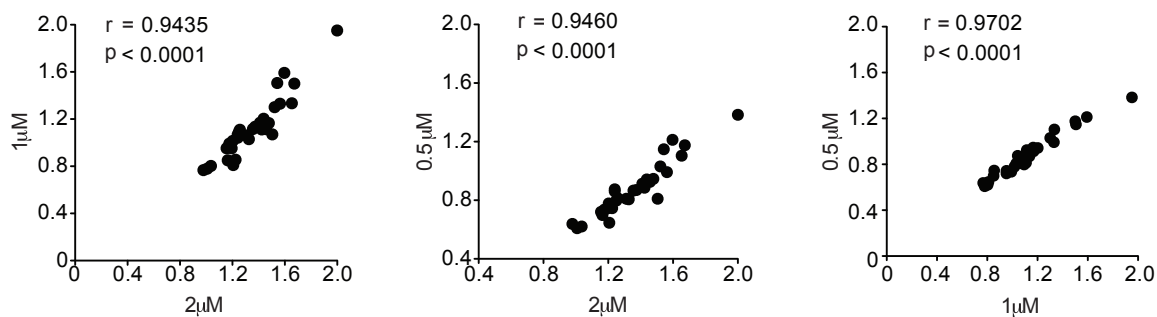

**B**

HLA-B alleles

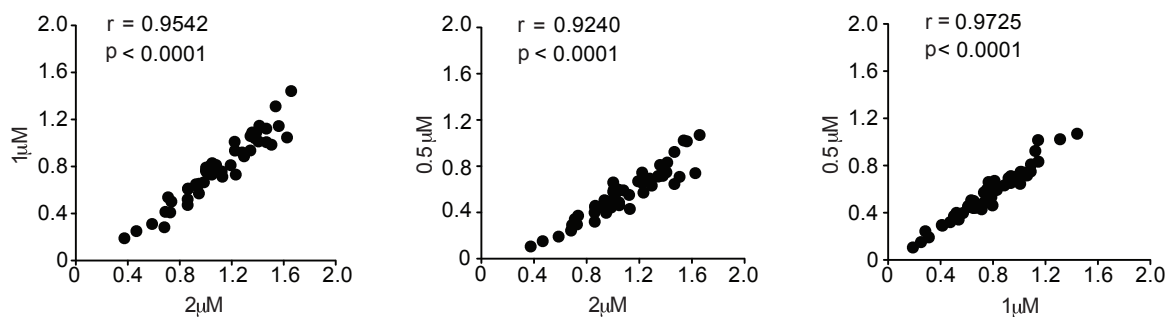

**C**

HLA-C alleles

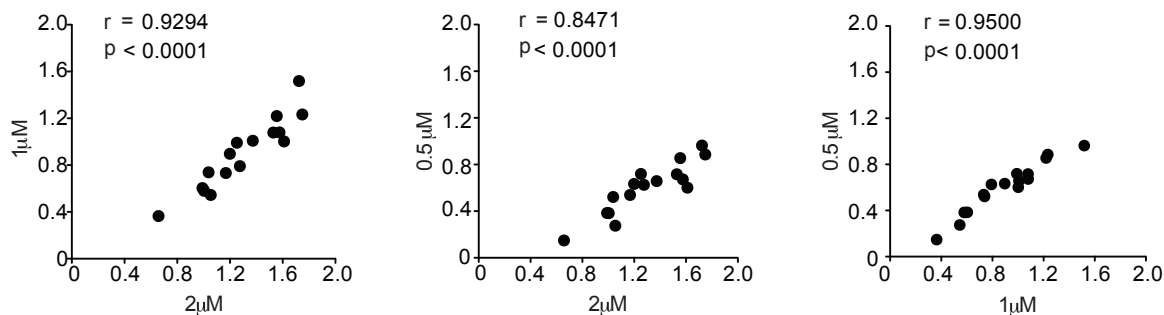

**D**

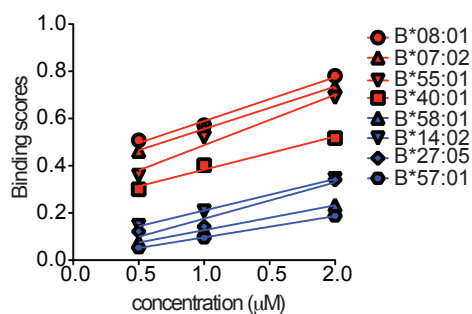

Supplement: Figure S4 — The relative LILR binding strength to different HLA allotypes was similar at each of the LILR concentrations tested. Spearman correlations between the binding scores of 3 different tested concentrations of LILRB2 to HLA-A (A), -B (B) and -C (C) allotypes. Spearman correlation coefficient and p values are indicated on graphs. (D) Analysis of LILRB2 binding scores at the concentrations of 0.5, 1 and 2 µM to protective (blue) and high risk (red) HLA-B allotypes. (PDF) [file pgen.1004196.s004.pdf]
