# Supplementary material for: LILRB2 Interaction with HLA Class I Correlates with Control of HIV-1 Infection
Source: PLoS Genet. 2014 Mar 6;10(3):e1004196. doi: 10.1371/journal.pgen.1004196 (PMC3945438; doi:10.1371/journal.pgen.1004196)
Supplement: Table S1 — HLA class I allele-specific LILRB1 and LILRB2 binding scores and corresponding odds ratios (OR) for viral load control as determined in a univariate model for each corresponding allele. ORs were not defined for some HLA alleles that cannot be genotyped (C*17:01 and C*18:02) and for alleles that were not present in the controller groups or in the whole population. Analysis for the combined cohort (All) was adjusted for race. (PDF) [file pgen.1004196.s005.pdf]

Table S1. *HLA* class I allele-specific LILRB1 and LILRB2 binding scores and corresponding odds ratios (OR) for viral load control as determined in a univariate model for each corresponding allele. ORs were not defined for some *HLA* alleles that cannot be genotyped (*C*17:01* and *C*18:02*) and for alleles that were not present in the controller groups or in the whole population. Analysis for the combined cohort (All) was adjusted for race.

	**Binding score**		**All (N=3991)**		**Whites (N=2685)**				**Blacks (N=1306)**			
	**LILRB1**	**LILRB2**	**OR**	**p**	**OR**	**p**	**Controllers (N=808)**	**Non-controllers (N=1877)**	**OR**	**p**	**Controllers (N=477)**	**Non-controllers (N=829)**
A*01:01	0.36	0.55	**1.3**	**4E-03**	**1.3**	**4E-03**	200	577	1.2	5E-01	37	76
A*02:01	0.11	0.60	**1.3**	**3E-04**	**1.3**	**2E-03**	382	1040	1.3	7E-02	102	213
A*02:03	0.17	0.38	1.0	1E+00	0.4	6E-01	1	1			0	1
A*02:06	0.28	0.52	**0.2**	**1E-03**	**0.2**	**4E-03**	12	6	0.2	2E-01	3	1
A*03:01	0.41	0.51	1.0	5E-01	1.1	6E-01	214	530	**0.7**	**4E-02**	95	129
A*11:01	0.48	0.48	**0.8**	**2E-02**	**0.8**	**4E-02**	128	236	0.7	3E-01	14	16
A*11:02	0.63	0.80										
A*23:01	0.38	0.51	**1.8**	**1E-05**	1.6	7E-02	21	76	**1.9**	**7E-05**	69	214
A*24:02	0.43	0.55	1.2	8E-02	1.2	1E-01	116	317	1.2	5E-01	20	43
A*24:03	0.41	0.98	0.5	3E-01	0.6	5E-01	4	6	0.0	1E+00	1	0
A*25:01	0.11	0.56	**0.5**	**2E-05**	**0.5**	**2E-05**	68	81	0.6	4E-01	5	5
A*26:01	0.11	0.67	1.0	1E+00	1.0	8E-01	51	115	1.2	6E-01	11	23
A*29:01	0.18	0.56	0.9	8E-01	1.0	1E+00	5	12	0.0	1E+00	1	0
A*29:02	0.13	0.53	1.2	3E-01	**1.5**	**5E-02**	38	130	0.8	4E-01	33	50
A*30:01	0.56	0.75	**0.7**	**2E-02**	**0.5**	**4E-03**	33	40	0.9	4E-01	54	81
A*30:02	0.21	0.40	**0.6**	**2E-03**	0.7	3E-01	18	30	**0.6**	**3E-03**	89	102
A*31:01	0.24	0.48	**0.5**	**3E-06**	**0.6**	**5E-04**	70	96	**0.4**	**1E-03**	30	21
A*32:01	0.10	0.58	**0.8**	**3E-02**	**0.7**	**2E-02**	87	146	1.0	9E-01	17	28
A*33:01	0.07	0.58	0.9	5E-01	0.6	2E-01	14	21	1.1	8E-01	15	28
A*33:03	0.12	0.66	1.0	9E-01	1.9	2E-01	6	26	0.9	4E-01	58	91
A*34:01	0.14	0.57	1.1	1E+00			0	1	0.6	7E-01	1	1
A*34:02	0.26	0.50	0.7	2E-01	0.4	3E-01	3	3	0.8	3E-01	38	53
A*36:01	0.20	0.40	**2.8**	**3E-03**	0.9	8E-01	3	6	**3.5**	**1E-03**	8	49
A*43:01	0.07	0.39										
A*66:01	0.26	0.75	1.3	3E-01	1.7	3E-01	6	23	1.1	7E-01	15	29

A*66:02	0.20	0.53	0.7	3E-01					0.7	3E-01	11	13
A*68:01	0.13	0.43	1.2	2E-01	1.1	5E-01	43	113	1.4	2E-01	27	63
A*68:02	0.11	0.43	1.0	8E-01	**0.5**	**2E-02**	27	32	1.3	2E-01	49	106
A*69:01	0.18	0.65	0.9	8E-01	1.0	1E+00	4	9	0.6	7E-01	1	1
A*74:01/2	0.04	0.53	**0.6**	**1E-02**	0.4	6E-01	1	1	**0.7**	**1E-02**	73	84
A*80:01	0.12	0.56	0.8	6E-01					0.8	6E-01	6	8
B*07:02	0.22	0.56	**2.2**	**7E-15**	**2.3**	**2E-12**	104	486	**1.9**	**7E-04**	45	135
B*08:01	0.38	0.57	**1.8**	**3E-07**	**2.0**	**1E-07**	96	392	1.2	5E-01	25	52
B*13:01	0.12	0.50	0.5	6E-01			0	1	0.0	1E+00	1	0
B*13:02	0.18	0.38	**0.6**	**3E-04**	**0.5**	**9E-04**	56	74	0.6	2E-01	13	13
B*14:01	0.27	0.40	0.8	2E-01	0.8	3E-01	25	45	0.7	4E-01	7	8
B*14:02	0.21	0.21	**0.5**	**3E-06**	**0.5**	**1E-05**	84	104	0.6	9E-02	30	33
B*15:01	0.31	0.44	1.0	8E-01	0.9	7E-01	97	219	1.2	7E-01	11	23
B*15:02	0.37	0.29	0.0	1E+00	0.0	1E+00	1	0				
B*15:03	0.35	0.49	1.2	3E-01	0.8	7E-01	4	8	1.2	2E-01	52	106
B*15:10	0.22	0.20	**2.1**	**3E-03**	0.9	9E-01	1	2	**2.2**	**3E-03**	20	73
B*15:11	0.27	0.47										
B*15:12	0.39	0.38										
B*15:13	0.19	0.37									0	1
B*15:16	0.27	0.31	0.8	6E-01			0	3	0.8	4E-01	20	27
B*18:01	0.40	0.66	**1.4**	**1E-02**	1.4	7E-02	56	171	1.7	6E-02	16	47
B*27:05	0.27	0.14	**0.3**	**1E-16**	**0.4**	**9E-15**	131	123	**0.3**	**4E-03**	17	9
B*27:08	0.49	0.30					0	1				
B*35:01	0.36	0.41	**1.6**	**8E-05**	**1.4**	**2E-02**	68	221	**1.9**	**9E-04**	39	122
B*38:01	0.30	0.39	1.5	7E-02	1.5	1E-01	27	86	2.0	4E-01	2	7
B*39:01	0.35	0.32	0.9	6E-01	1.0	9E-01	23	50	0.4	3E-01	4	3
B*40:01	0.18	0.40	**1.7**	**6E-04**	**2.0**	**5E-05**	42	190	0.7	3E-01	13	16
B*40:02	0.23	0.33	**0.6**	**4E-03**	**0.6**	**9E-03**	37	49	0.5	3E-01	5	4
B*40:06	0.24	0.51	0.0	1E+00	0.0	1E+00	1	0	0.0	1E+00	3	0
B*41:01	0.39	0.39	1.1	8E-01	1.0	9E-01	8	18	1.3	7E-01	4	9
B*42:01	0.42	0.38	1.1	6E-01	0.4	4E-01	2	2	1.2	5E-01	37	77
B*44:02	0.19	0.36	1.2	2E-01	1.2	1E-01	121	325	0.8	6E-01	17	25
B*44:03	0.19	0.16	**1.3**	**5E-02**	**1.6**	**7E-03**	56	185	0.9	8E-01	49	82
B*45:01	0.45	0.54	**2.6**	**5E-05**	1.0	9E-01	9	20	**3.6**	**7E-06**	15	89
B*46:01	0.31	0.57										
B*48:01	0.14	0.52	0.2	9E-02	0.3	2E-01	4	2	0.0	1E+00	1	0

B*49:01	0.06	0.24	1.4	7E-02	1.5	1E-01	21	70	1.3	3E-01	21	49
B*50:01	0.26	0.32	1.4	2E-01	1.4	3E-01	13	43	1.5	5E-01	7	15
B*51:01	0.18	0.46	1.0	1E+00	1.0	7E-01	83	185	1.2	6E-01	20	43
B*51:02	0.23	0.33	0.0	1E+00	0.0	1E+00	1	0	0.0	1E+00	1	0
B*52:01	0.10	0.54	**0.5**	**2E-04**	**0.4**	**2E-04**	39	40	0.7	1E-01	22	27
B*53:01	0.36	0.47	**1.3**	**4E-02**	0.8	4E-01	12	21	**1.5**	**1E-02**	84	198
B*54:01	0.25	0.26										
B*55:01	0.21	0.52	**2.1**	**2E-02**	**2.1**	**3E-02**	10	49	2.0	4E-01	2	7
B*56:01	0.25	0.27	1.8	1E-01	1.7	2E-01	7	29	2.3	5E-01	1	4
B*57:01	0.07	0.09	**0.2**	**9E-35**	**0.2**	**2E-34**	205	132	0.4	5E-02	13	10
B*57:03	0.26	0.25	**0.1**	**3E-31**	**0.1**	**5E-08**	29	6	**0.2**	**3E-24**	131	45
B*58:01	0.03	0.12	**0.6**	**8E-03**	**0.5**	**6E-03**	26	29	0.8	2E-01	39	56
B*59:01	0.18	0.37										
B*67:01	0.47	0.41									0	1
B*73:01	0.33	0.53	0.2	2E-01	0.2	2E-01	2	1				
B*78:01	0.34	0.72	3.3	6E-02			0	1	3.1	7E-02	3	16
B*81:01	0.32	0.50	**0.2**	**5E-09**	0.0	1E+00	1	0	**0.2**	**9E-09**	55	24
B*82:01	0.36	0.41									0	7
C*01:02	0.04	0.39	**0.6**	**5E-04**	**0.6**	**1E-04**	96	138	1.2	6E-01	9	19
C*02:02	0.19	0.54	**0.7**	**1E-02**	**0.6**	**7E-04**	102	158	1.3	3E-01	23	52
C*03:02	0.13	0.29	1.2	5E-01	0.9	9E-01	6	13	1.3	4E-01	15	35
C*03:03	0.20	0.30	1.1	5E-01	1.2	3E-01	59	162	0.8	5E-01	11	15
C*03:04	0.39	0.37	**1.5**	**7E-04**	**1.5**	**5E-03**	85	281	1.5	5E-02	39	99
C*04:01	0.04	0.18	**1.7**	**1E-09**	**1.8**	**3E-07**	123	476	**1.5**	**6E-04**	139	338
C*05:01	0.24	0.49	1.2	6E-02	**1.3**	**2E-02**	106	320	0.9	6E-01	27	41
C*06:02	0.31	0.76	**0.6**	**5E-12**	**0.4**	**7E-22**	292	330	**1.5**	**2E-02**	64	153
C*07:02	0.15	0.62	**2.1**	**8E-14**	**2.2**	**7E-12**	122	530	**1.8**	**2E-03**	39	118
C*08:01	0.22	0.36	**0.3**	**3E-02**	0.3	5E-02	7	4	0.3	3E-01	2	1
C*12:03	0.21	0.54	**0.7**	**1E-02**	0.9	2E-01	98	200	**0.5**	**3E-03**	37	29
C*14:02	0.16	0.5	**0.6**	**2E-03**	**0.5**	**1E-03**	37	40	0.7	3E-01	23	30
C*15:02	0.36	0.45	0.7	6E-02	**0.7**	**3E-02**	51	75	1.2	8E-01	8	14
C*16:01	0.14	0.50	**1.6**	**1E-04**	1.3	1E-01	42	124	**1.9**	**2E-04**	56	168
C*17:01	0.08	0.27										
C*18:02	0.29	0.61										
