# Supplementary material for: LILRB2 Interaction with HLA Class I Correlates with Control of HIV-1 Infection
Source: PLoS Genet. 2014 Mar 6;10(3):e1004196. doi: 10.1371/journal.pgen.1004196 (PMC3945438; doi:10.1371/journal.pgen.1004196)
Supplement: Table S2 — Spearman correlation between LILRB1 binding strength and odds ratios (p<0.05) for viral load control in HIV-1-infected individuals. (PDF) [file pgen.1004196.s006.pdf]

Table S2. Spearman correlation between LILRB1 binding strength and odds ratios ($p<0.05$) for viral load control in HIV-1-infected individuals.

	N (alleles)	r	p
**Whites (N=2685)**			
HLA-A	10	-0.05	0.9
HLA-B	14	0.20	0.5
HLA-C	9	-0.17	0.7
**Blacks (N=1306)**			
HLA-A	6	0.03	1.0
HLA-B	8	0.29	0.5
HLA-C	5	-0.60	0.3
