# Supplementary material for: LILRB2 Interaction with HLA Class I Correlates with Control of HIV-1 Infection
Source: PLoS Genet. 2014 Mar 6;10(3):e1004196. doi: 10.1371/journal.pgen.1004196 (PMC3945438; doi:10.1371/journal.pgen.1004196)
Supplement: Table S3 — Effect of the LILRB2-HLA binding strength and individual class I alleles on mVL in white patients. Linear regression models with stepwise selection included all HLA class I alleles with phenotypic frequencies of >2% and one of the A, B, C or ABC binding scores at a time. The results are shown for the p<0.05 cut-off. The A and C scores did not stay in the model. (PDF) [file pgen.1004196.s007.pdf]

Table S3. Effect of the LILRB2-HLA binding strength and individual class I alleles on mVL in white patients. Linear regression models with stepwise selection included all *HLA* class I alleles with phenotypic frequencies of >2% and one of the A, B, C or ABC binding scores at a time. The results are shown for the p<0.05 cut-off. The A and C scores did not stay in the model.

Whites (N=2900)							
**Covariate**	**Δ[1]**	**SE**	**p**	**Covariate**	**Δ[1]**	**SE**	**p**
B*57:01	-0.57	0.07	9E-15	B*57:01	-0.62	0.07	6E-20
**LILRB2-B[2]**	**0.09**	**0.01**	**4E-11**	A*01:01	0.29	0.04	2E-11
C*12:02	-0.65	0.11	7E-10	C*12:02	-0.62	0.11	3E-09
A*25:01	-0.45	0.07	8E-10	**LILRB2-ABC[2]**	**0.08**	**0.01**	**4E-09**
A*01:01	0.25	0.04	5E-09	A*25:01	-0.40	0.07	8E-08
B*13:02	-0.43	0.08	2E-08	C*14:02	-0.48	0.10	2E-06
C*14:02	-0.49	0.10	9E-07	B*49:01	0.47	0.11	9E-06
A*31:01	-0.32	0.07	3E-06	B*13:02	-0.37	0.09	2E-05
B*49:01	0.49	0.11	4E-06	B*40:01	0.26	0.06	4E-05
C*07:01	-0.20	0.05	2E-05	B*27:05	-0.27	0.07	1E-04
A*32:01	-0.23	0.06	1E-04	A*32:01	-0.22	0.06	3E-04
B*40:01	0.24	0.06	1E-04	A*31:01	-0.23	0.07	1E-03
B*27:05	-0.25	0.07	7E-04	C*04:01	0.15	0.05	1E-03
C*15:02	-0.27	0.08	9E-04	C*07:01	-0.15	0.05	2E-03
B*15:01	-0.17	0.05	2E-03	C*15:02	-0.26	0.08	2E-03
C*08:02	-0.19	0.06	4E-03	B*44:03	0.22	0.07	3E-03
B*44:03	0.21	0.07	4E-03	A*30:01	-0.32	0.12	7E-03
A*11:01	-0.13	0.05	2E-02	B*15:01	-0.14	0.05	8E-03
C*04:01	0.11	0.04	2E-02	C*08:02	-0.17	0.06	1E-02
				A*68:01	0.19	0.08	1E-02
				B*07:02	0.11	0.05	3E-02
				A*24:02	0.10	0.05	3E-02

[1] change in log10 viral load due to the presence of the corresponding *HLA* allele or for the increase by 0.1 unit of binding score

[2] did not stay in the model with the p<0.01 or p<0.001 cut-offs
