# Supplementary material for: LILRB2 Interaction with HLA Class I Correlates with Control of HIV-1 Infection
Source: PLoS Genet. 2014 Mar 6;10(3):e1004196. doi: 10.1371/journal.pgen.1004196 (PMC3945438; doi:10.1371/journal.pgen.1004196)
Supplement: Table S4 — Effect of the LILRB2-HLA binding strength and individual class I alleles on mVL in black patients. The analysis was similar to the one described in Table S3. The results are shown for the p<0.05 cut-off. The A and C scores did not stay in the model. (PDF) [file pgen.1004196.s008.pdf]

Table S4. Effect of the LILRB2-HLA binding strength and individual class I alleles on mVL in black patients. The analysis was similar to the one described in Table S3. The results are shown for the p<0.05 cut-off. The A and C scores did not stay in the model.

**Blacks (n=1490)**							
**Covariate**	**Δ[1]**	**SE**	**p**	**Covariate**	**Δ[1]**	**SE**	**p**
B*57:03	-0.71	0.07	2E-25	B*57:03	-0.64	0.07	8E-19
B*81:01	-0.54	0.11	4E-07	A*23:01	0.28	0.05	2E-07
**LILRB2-B[2]**	**0.06**	**0.01**	**1E-06**	B*58:02	0.40	0.09	4E-06
B*58:02	0.42	0.09	2E-06	A*36:01	0.45	0.11	3E-05
B*15:10	0.41	0.09	7E-06	B*52:01	-0.48	0.12	4E-05
A*23:01	0.23	0.05	2E-05	B*15:10	0.38	0.09	4E-05
C*12:03	-0.40	0.10	5E-05	C*12:03	-0.40	0.10	5E-05
B*35:01	0.23	0.07	7E-04	C*16:01	0.22	0.06	7E-04
A*36:01	0.37	0.11	7E-04	B*81:01	-0.39	0.12	1E-03
B*45:01	0.26	0.08	1E-03	**LILRB2-ABC[3]**	**0.03**	**0.01**	**1E-03**
B*52:01	-0.32	0.11	3E-03	A*01:01	0.23	0.08	3E-03
A*03:01	-0.14	0.06	1E-02	C*08:04	-0.39	0.14	8E-03
A*74:01/2	-0.16	0.07	1E-02	B*35:01	0.17	0.07	9E-03
C*08:04	-0.32	0.14	2E-02	B*14:02	-0.26	0.10	1E-02
C*07:01	0.12	0.05	3E-02	B*57:01	-0.39	0.15	1E-02
A*24:02	0.21	0.10	3E-02	A*24:02	0.23	0.10	2E-02
B*14:02	-0.21	0.10	4E-02	C*18:00	-0.17	0.08	4E-02
A*11:01	-0.26	0.13	4E-02	A*74:01/2	-0.13	0.06	5E-02

[1] change in log10 viral load due to the presence of an *HLA* allele or for the increase by 0.1 unit of binding score

[2] stayed in the model with the p<0.01 and p<0.001 cut-offs

[3] stayed in the model with the p<0.01 cut-off and not with the p<0.001cut-off
