# Supplementary material for: LILRB2 Interaction with HLA Class I Correlates with Control of HIV-1 Infection
Source: PLoS Genet. 2014 Mar 6;10(3):e1004196. doi: 10.1371/journal.pgen.1004196 (PMC3945438; doi:10.1371/journal.pgen.1004196)
Supplement: Table S5 — Effect of individual class I alleles on viral control (controllers vs. noncontrollers). Logistic regression model with stepwise selection included all HLA class I alleles with phenotypic frequencies of >2%. The results are shown for the p<0.05 cut-off. (PDF) [file pgen.1004196.s009.pdf]

Table S5. Effect of individual class I alleles on viral control (controllers vs. non-controllers). Logistic regression model with stepwise selection included all *HLA* class I alleles with phenotypic frequencies of >2%. The results are shown for the p<0.05 cut-off.

**Whites (N=2685)**			
	**p**	**OR**	**95%CI**
B*57:01	3E-42	0.1	0.1-0.2
B*27:05	3E-13	0.3	0.2-0.4
A*01:01	2E-11	2.3	1.8-2.9
B*07:02	2E-07	2.0	1.5-2.6
B*52:01	4E-06	0.3	0.2-0.5
B*14:02	2E-05	0.5	0.3-0.7
B*13:02	3E-05	0.4	0.3-0.6
A*02:01	4E-05	1.5	1.2-1.8
C*14:02	1E-04	0.4	0.2-0.6
B*40:01	4E-04	2.0	1.4-2.9
B*38:01	5E-04	2.9	1.6-5.2
B*18:01	7E-04	2.0	1.3-3.0
C*04:01	1E-03	1.5	1.2-2.0
A*25:01	1E-03	0.5	0.3-0.8
B*40:02	4E-03	0.5	0.3-0.8
A*31:01	5E-03	0.6	0.4-0.9
C*12:03	1E-02	0.6	0.4-0.9
B*58:01	2E-02	0.5	0.3-0.9
A*68:02	3E-02	0.5	0.3-0.9
B*35:02	3E-02	2.9	1.1-7.9
B*55:01	4E-02	2.1	1.0-4.4

**Blacks (N=1306)**			
	**p**	**OR**	**95%CI**
B*57:03	2E-22	0.1	0.1-0.2
C*12:03	8E-05	0.3	0.2-0.6
B*45:01	8E-05	3.3	1.8-5.9
A*23:01	3E-04	1.9	1.3-2.7
B*35:01	3E-04	2.2	1.4-3.3
A*36:01	4E-04	4.3	1.9-9.8
B*15:10	1E-03	2.6	1.5-4.7
B*81:01	2E-03	0.4	0.2-0.7
B*58:02	3E-03	2.5	1.4-4.5
C*08:04	4E-03	0.3	0.1-0.7
B*18:01	5E-03	2.5	1.3-4.8
B*07:02	1E-02	1.7	1.1-2.5
C*05:01	2E-02	0.5	0.3-0.9
A*03:01	4E-02	0.7	0.5-1.0
B*14:02	4E-02	0.6	0.3-1.0
