# Supplementary material for: LILRB2 Interaction with HLA Class I Correlates with Control of HIV-1 Infection
Source: PLoS Genet. 2014 Mar 6;10(3):e1004196. doi: 10.1371/journal.pgen.1004196 (PMC3945438; doi:10.1371/journal.pgen.1004196)
Supplement: Table S6 — Effect of individual HLA class I alleles on mVL. Linear regression model with stepwise selection included all HLA class I alleles with phenotypic frequencies of >2%. The results are shown for the p<0.05 cut-off. (PDF) [file pgen.1004196.s010.pdf]

Table S6. Effect of individual *HLA* class I alleles on mVL. Linear regression model with stepwise selection included all *HLA* class I alleles with phenotypic frequencies of >2%. The results are shown for the p<0.05 cut-off.

Whites (N=2900)				Blacks (N=1490)			
**Allele**	**Δ**	**SE**	**p**	**Allele**	**Δ**	**SE**	**p**
B*57:01	-0.82	0.05	1E-51	B*57:03	-0.72	0.06	2E-29
B*27:05	-0.46	0.06	2E-14	B*58:02	0.44	0.09	5E-07
A*01:01	0.27	0.04	5E-11	A*23:01	0.25	0.05	3E-06
B*13:02	-0.41	0.08	1E-07	B*81:01	-0.46	0.11	1E-05
B*07:02	0.24	0.04	1E-07	C*12:03	-0.38	0.10	1E-04
A*25:01	-0.39	0.08	5E-07	A*36:01	0.40	0.11	2E-04
B*14:02	-0.34	0.07	1E-06	B*15:10	0.32	0.09	3E-04
C*03:04	0.26	0.06	2E-06	B*35:01	0.22	0.07	1E-03
C*12:02	-0.49	0.10	2E-06	A*01:01	0.25	0.08	1E-03
B*15:01	-0.31	0.07	4E-06	B*52:01	-0.36	0.12	3E-03
A*31:01	-0.31	0.07	7E-06	B*57:01	-0.45	0.15	3E-03
C*14:02	-0.42	0.10	3E-05	B*14:02	-0.30	0.10	3E-03
A*32:01	-0.23	0.06	2E-04	B*18:01	0.27	0.10	8E-03
C*03:03	0.26	0.08	6E-04	B*45:01	0.24	0.10	1E-02
B*58:01	-0.40	0.12	6E-04	B*07:02	0.15	0.06	2E-02
C*04:01	0.15	0.05	7E-04	C*08:04	-0.30	0.14	3E-02
B*49:01	0.23	0.10	1E-02	C*16:01	0.16	0.07	4E-02
A*11:01	-0.13	0.05	2E-02	A*24:02	0.21	0.10	4E-02
B*40:02	-0.23	0.10	2E-02	A*03:01	-0.12	0.06	4E-02
C*15:02	-0.18	0.08	3E-02	A*74:01/2	-0.13	0.06	4E-02
B*18:01	0.15	0.07	3E-02				
